# Construction and exploratory validation of a digital pathology prognostic model for stage II–III colorectal cancer based on contrastive learning and attention mechanisms

**DOI:** 10.3389/fonc.2026.1863967

**Published:** 2026-08-27

**Authors:** Yizhan Lu, Zhifeng Qu, Chongbao Sun, Bo Li, Xiaoyang Bai, Qingqing Zhao, Wenxin Zhang, Yandong Zhao, Wuteng Cao, Xuezhi Zhou

**Affiliations:** 1 Department of Medical Equipment, The First Affiliated Hospital, and College of Clinical Medicine of Henan University of Science and Technology, Luoyang, China; 2 Department of Radiology, The Sixth Affiliated Hospital, Sun Yat-sen University, Guangzhou, Guangdong, China; 3 Guangdong Provincial Key Laboratory of Colorectal and Pelvic Floor Diseases, The Sixth Affiliated Hospital, Sun Yat-sen University, Guangzhou, Guangdong, China; 4 Biomedical Innovation Center, The Sixth Affiliated Hospital, Sun Yat-sen University, Guangzhou, Guangdong, China; 5 College of Medical Engineering, Henan Medical University, Xinxiang, Henan, China; 6 Department of Pathology, The Sixth Affiliated Hospital, Sun Yat-sen University, Guangzhou, Guangdong, China; 7 College of Mathematical Medicine, Henan Medical University, Xinxiang, Henan, China; 8 Xinxiang City Engineering Technology Research Center of Intelligent Brain Imaging and Brain Modulation, Xinxiang, Henan, China

**Keywords:** colorectal cancer, contrastive learning, prognostic prediction, progression-free survival, whole-slide image

## Abstract

**Introduction:**

Although TNM staging is the standard framework for prognostic assessment in colorectal cancer (CRC), patients within the same stage can have heterogeneous outcomes. This study aimed to extract tumor-tissue features from whole-slide images (WSIs), develop an image-derived prognostic model for stage II–III CRC, and conduct an exploratory evaluation of its performance.

**Methods:**

A transfer-learning tissue classifier was used to identify tumor regions in CRC WSIs. Self-supervised contrastive learning was used to extract tumor features, which were summarized by patient-level K-Means clustering. An attention-based survival model generated a locked image-derived Risk Score. The actual analyzed population comprised 335 patients with locally advanced CRC: 318 patients recorded as stage III and 17 patients with T4N0 disease (high-risk stage II by AJCC eighth-edition anatomic staging). The 265-patient TCGA cohort was randomly divided 8:2 into a training set and an internal holdout set; 70 SAHSYU patients formed a single-center exploratory cohort. Discrimination was quantified with patient-level bootstrap confidence intervals. Cox regression, Kaplan–Meier analysis, one-year calibration, and exploratory decision-curve analysis were performed. A sensitivity analysis excluded all N0 patients without retraining or changing the locked Risk Score.

**Results:**

The C-index was 0.704 (95% bootstrap CI 0.637–0.768) in training, 0.663 (0.544–0.776) in the internal holdout set, and 0.706 (0.533–0.859) in SAHSYU. Per one training-set standard deviation, the Risk Score was associated with PFS internally (HR 1.688, 95% CI 1.080–2.640; Wald p=0.022) and exploratorily in SAHSYU (HR 6.010, 95% CI 1.170–30.877; p=0.032). In the sensitivity analysis excluding all N0 records without retraining, the C-index remained 0.647 internally and 0.705 in SAHSYU; fixed-threshold log-rank p values were 0.014 and 0.027, respectively.

**Conclusion:**

The image-derived Risk Score showed preliminary PFS discrimination and risk-stratification potential in stage II–III CRC. These findings require validation in larger multicenter cohorts with more events, longer follow-up, formal calibration, and clinical-utility assessment before treatment decisions can be informed.

## 1 Introduction

Colorectal cancer (CRC) is one of the most common cancers worldwide. According to the American Cancer Society, an estimated 154,270 new CRC cases and 52,900 deaths were expected in the United States in 2025, making CRC the third most common cancer and the second leading cause of cancer-related death (1). The tumor–node–metastasis (TNM) staging system is the principal framework for risk stratification and prognostic assessment (2). Based on evidence reflected in European Society for Medical Oncology (ESMO) (3) and National Comprehensive Cancer Network (NCCN) (4) guidance, patients with stage III colon cancer are commonly categorized as lower risk (T1–3N1) or higher risk (T4 and/or N2) when the duration of adjuvant chemotherapy is considered. Nevertheless, outcomes remain heterogeneous among patients with similar stage and treatment (5–7), and risk assessment is also challenging in high-risk stage II disease (8). TNM staging captures anatomic extent but does not fully represent tumor biology. Molecular testing can add information but may be costly or inaccessible in some settings. An objective and scalable tool derived from routine pathology could therefore complement, established clinicopathological assessment in locally advanced CRC.

The development of digital pathology has enabled complete digitization of hematoxylin-and-eosin (H&E)-stained whole-slide images (WSIs) (9). WSIs contain prognostically relevant information on tumor architecture, stroma, immune-cell-rich regions, mucin, necrosis, and other morphological components (10). Deep learning can extract high-dimensional representations from WSIs and has shown potential for outcome modeling (11–13). Previous CRC studies used transfer learning or supervised models to construct prognostic biomarkers (14, 15). However, supervised approaches can require costly manual annotation, and many models remain difficult to interpret or transport across centers.

Contrastive learning is a self-supervised strategy that can learn discriminative representations without outcome or region annotations during pretraining (16). It has been applied to prognostic and biological tasks in computational pathology. Fuster et al. used self-contrastive weak supervision for WSI-based prognosis (17); Zhang et al. developed a contrastive clustering-derived pathological signature in small-cell lung cancer (18); and Huang et al. proposed HistCode for tumor diagnosis and gene-expression prediction (19).

Attention mechanisms dynamically allocate weights to input features and have become widely used for adaptive feature aggregation (20, 21). In digital pathology, attention weights can help localize image regions that influence a model output. Attention-based multiple-instance learning, CLAM, and transformer-based aggregation such as TransMIL provide alternative mechanisms for combining patch-level representations into slide-level predictions (22–24). The present study evaluated a specific integration of annotation-free feature learning, patient-level phenotypic clustering, attention-weighted survival modeling, and spatial projection of model weights in a locally advanced CRC workflow.

This study aimed to construct an exploratory digital-pathology prognostic framework that integrated self-supervised contrastive learning, patient-level clustering, and attention-based survival modeling. Tumor features extracted from routine H&E WSIs were used to generate a Risk Score, evaluate preliminary PFS discrimination and risk stratification, and visualize spatially weighted phenotypic regions.

## 2 Materials and methods

### 2.1 Data acquisition

This retrospective study was approved by the Ethics Committee of the Sixth Affiliated Hospital of Sun Yat-sen University (SAHSYU), which waived the requirement for informed consent. A total of 335 patients with locally advanced CRC were included. 318 patients recorded as stage III and 17 patients recorded as T4N0 (15 in TCGA and 2 in SAHSYU), which correspond to high-risk stage II disease under AJCC eighth-edition anatomic staging. The TCGA modeling cohort comprised 265 patients with postoperative H&E WSIs and 100 recorded PFS events; the SAHSYU exploratory cohort comprised 70 patients and 10 PFS events. Clinical characteristics and follow-up information were collected. PFS was defined as the interval from initiation of postoperative treatment to tumor progression or death from any cause. Patients without postoperative H&E WSIs, survival information, or complete clinicopathological characteristics were excluded.

The training and validation of the tissue classification model were conducted using the publicly available datasets NCT-CRC-HE-100K and NCT-CRC-HE-7K, which were developed by Kather et al. (14). The NCT-CRC-HE-100K dataset comprises nearly 100,000 224×224pixel image tiles generated from pathological sections of 86 CRC patients, intended for model training. The NCT-CRC-HE-7K dataset comprises 7,180 image tiles generated from tissue sections of 25 CRC patients, serving to validate model performance. Both datasets were annotated with nine tissue types: adipose (ADI), background (BAC), debris (DEB), lymphocyte (LYM), mucus (MUC), smooth muscle (MUS), normal colonic mucosa (NOR), cancer-associated stroma (STR), and CRC tumor epithelium (TUM).

### 2.2 Pathological image preprocessing and tumor tissue extraction

All WSIs were divided into non-overlapping 224×224-pixel tiles at 20× magnification (0.5 μm/pixel). Macenko stain normalization, implemented with the Python StainTools package, was applied to reduce variation associated with staining and scanning.

**Figure 1:**
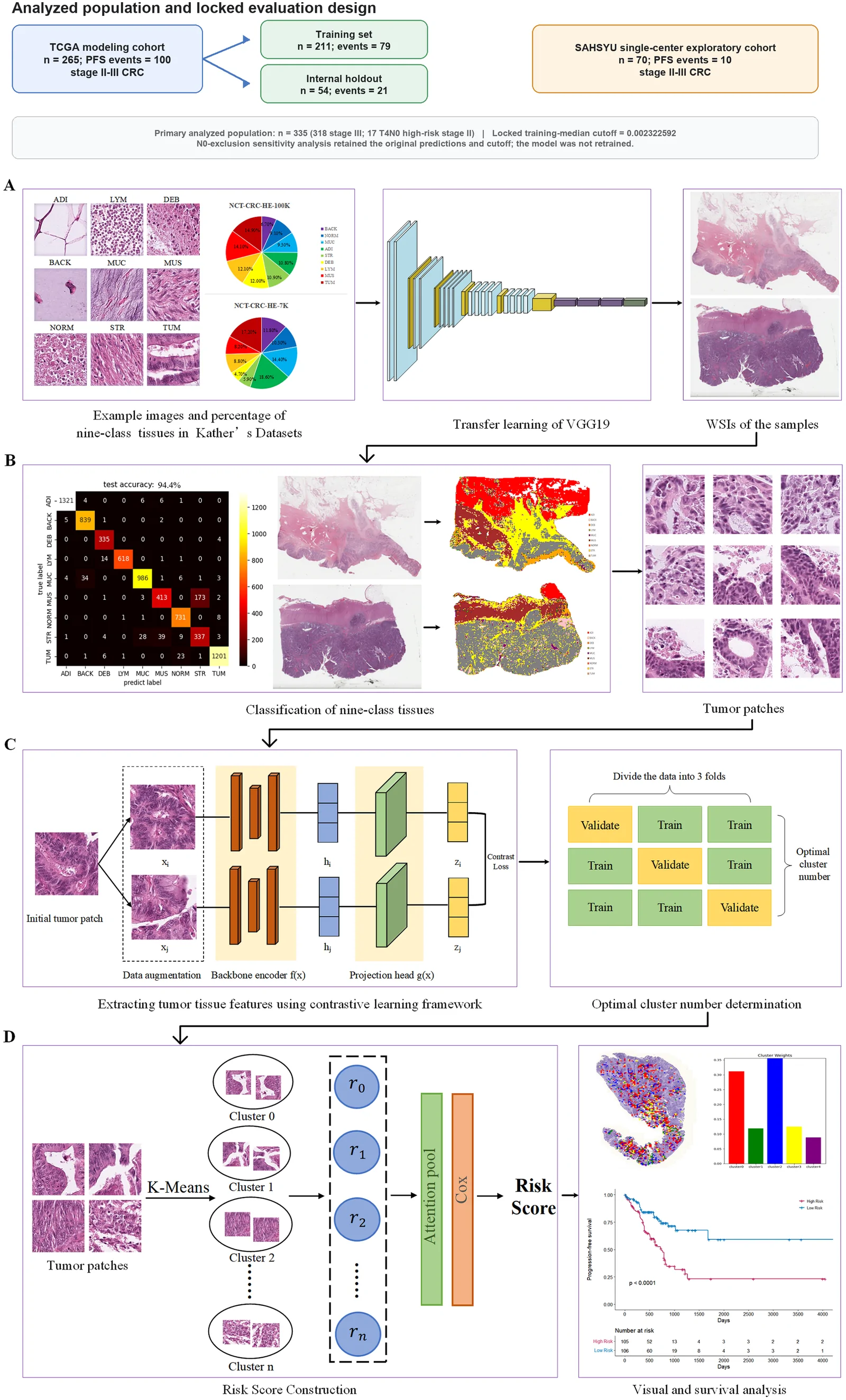
Study population and deep-learning workflow. The upper banner summarizes the analyzed population and locked evaluation design. The TCGA modeling cohort included 265 patients with stage II-III colorectal cancer (CRC) and 100 progression-free survival (PFS) events and was divided 8:2 into a training set (n = 211; 79 events) and an internal holdout set (n = 54; 21 events). The SAHSYU cohort included 70 patients and 10 PFS events and was used as a single-center exploratory evaluation. The primary analyzed population comprised 318 patients recorded as stage III and 17 patients with T4N0 high-risk stage II disease. **(A)** An ImageNet-pretrained VGG19 model was adapted for nine-class tissue classification. **(B)** The trained classifier was applied to TCGA and SAHSYU whole-slide images (WSIs), and tiles classified as tumor epithelium were retained. **(C)** Tumor-tile representations were learned using a SimCLR-style contrastive-learning framework with DenseNet121, followed by selection of five patient-level phenotypic clusters. **(D)** Cluster representations were aggregated by an attention-based survival network to generate the Risk Score. The training-set median cutoff (0.002322592) was locked for all subsequent analyses. The N0-exclusion sensitivity analysis retained the original predictions and cutoff without model retraining. ADI, adipose; BACK, background; DEB, debris; LYM, lymphocyte; MUC, mucus; MUS, smooth muscle; NORM, normal colonic mucosa; STR, cancer-associated stroma; TUM, CRC tumor epithelium; SAHSYU, Sixth Affiliated Hospital of Sun Yat-sen University; TCGA, The Cancer Genome Atlas.

The study workflow is shown in **Figure 1**. A nine-class tissue classifier was constructed using an ImageNet-pretrained VGG19 network and transfer learning (**Figure 1A**). Convolutional weights were retained, whereas the top fully connected and classification layers were replaced. The classifier was trained with the Adam optimizer (initial learning rate 1×10^-4^) and cross-entropy loss. Each tile was assigned the class with the highest predicted probability. The model achieved 94.4% accuracy on the tissue-classification validation set (**Figure 1B**). It was then applied to the TCGA and SAHSYU WSIs, and tiles classified as tumor epithelium (TUM) were retained for feature extraction.

### 2.3 Feature extraction from tumor tissue based on contrastive learning

A SimCLR-style contrastive learning framework with DenseNet121 as the backbone encoder was used to construct the feature extractor (**Figure 1C**). Tumor tiles were resized to 224×224 pixels. For each tile, an augmented view was generated using random rotation up to 20°, horizontal and vertical shifts of 0.2 of image width or height, shear of 0.2, zoom of 0.2, random horizontal flipping, and nearest-neighbor filling. The original and augmented views formed positive pairs; views from other images in the batch served as negatives.

DenseNet121 initialized with ImageNet weights served as the frozen backbone, followed by global average pooling and a 128-dimensional projection; the contrastive temperature was 0.12. Contrastive training used batch size 64. The survival network used a 128-dimensional input, 64-unit attention hidden layer, dropout 0.5, Adam learning rate 1 × 10^-4^, batch size 16. After training, 128-dimensional representations were extracted for the tumor tiles.

### 2.4 Construction of a prognostic model based on clustering and attention mechanisms

K-Means clustering was applied separately to the tumor-tile representations of each patient; TCGA and SAHSYU samples were not pooled for clustering. For each candidate number of clusters (K = 2–9), patient-level representations were generated and the mean C-index from three-fold cross-validation in the modeling workflow was used to select K. Five clusters yielded the highest archived mean cross-validation C-index (0.6449). For each patient, tumor tiles were assigned to five phenotypic clusters and the features within each cluster were aggregated to generate a patient-level matrix.

An attention-based survival network was constructed (**Figure 1D**). The five 128-dimensional cluster representations were passed to a two-layer attention module with a 64-unit hidden layer. Softmax-normalized attention weights were used to aggregate the cluster representations, and a fully connected network generated the Risk Score. The survival network was optimized using Cox partial log-likelihood loss, Adam (learning rate 1×10^-4^), dropout 0.5, batch size 16.

The TCGA modeling cohort was randomly divided 8:2 into a training set (211 patients)and an internal validation set (54 patients). Because SAHSYU was small, single-center, and event-sparse, it was conservatively described as a single-center exploratory evaluation.

### 2.5 Survival analysis and stratified analysis

The median Risk Score in the training set was used as the prespecified cutoff to classify patients as high or low risk. This cutoff was applied unchanged to the internal validation and SAHSYU cohorts. Kaplan–Meier curves were estimated and compared with two-sided log-rank tests.

Cox proportional hazards models were used to examine associations between the continuous Risk Score and PFS. Risk Score values were standardized using the training-set mean and standard deviation so that HRs represented a one-standard-deviation increase. Univariable models were fitted in the internal holdout and SAHSYU cohorts. An exploratory adjusted model including Risk Score, age, sex, T4, and N2 was fitted internally. No external multivariable model was fitted because only 10 PFS events were available. A sensitivity analysis excluded all N0 patients from the training, internal, and SAHSYU risk-score files while retaining the originally locked predictions and training-set median threshold; the image model was not retrained after this exclusion.

### 2.6 Statistical analysis

Statistical analysis and deep learning model construction were performed using R 4.1.3 software and the PyTorch 1.1 framework. Harrell’s C-index was used to assess discrimination (25), and patient-level nonparametric bootstrap resampling (2,000 replicates) provided 95% confidence intervals. Cox-model HRs, CIs, and p values were reported consistently with the two-sided Wald framework. Kaplan–Meier curves used the locked training-set median threshold and were compared with log-rank tests. Median follow-up was estimated with the reverse Kaplan–Meier method. One-year calibration was assessed in three approximately equal-sized groups in the internal holdout set.

## 3 Results

### 3.1 Patients characteristics

The demographic and clinicopathological characteristics are summarized in **Table 1**. The TCGA cohort contained 265 patients and 100 PFS events; the SAHSYU cohort contained 70 patients and 10 events. Fifteen TCGA and two SAHSYU patients were T4N0 and were classified as high-risk stage II. The reverse Kaplan–Meier median follow-up was 325 days in SAHSYU.

**Table 1:** Demographic and clinicopathological characteristics of the stage II–III TCGA and SAHSYU cohorts.

	TCGA cohort (100/265 events/patients)	SAHSYU cohort (10/70 events/patients)
Age		
≤60 year	99(37.4%)	33(47.1%)
>60 year	166(62.6%)	37(52.9%)
Sex		
Male	136(51.3%)	53(75.7%)
Female	129(48.7%)	17(24.3%)
T-stage		
T1	2(0.7%)	0
T2	13(4.9%)	1(1.5%)
T3	191(72.1%)	33(47.1%)
T4	59(22.3%)	36(51.4%)
N-stage		
N0 (all T4; high-risk stage II)	15(5.7%)	2(2.9%)
N1	141(53.2%)	40(57.1%)
N2	109(41.1%)	28(40.0%)

a Numbers in parentheses are PFS events/total patients. All 17 N0 records were T4N0 and were classified as high-risk stage II under AJCC eighth-edition anatomic staging.

### 3.2 Optimal cluster number determination and risk score construction

The contrastive learning network generated 128-dimensional tumor-tile features. In the archived three-fold evaluation across K = 2–9, five clusters yielded the highest mean C-index (0.6449) and were used in the subsequent exploratory model.

K-Means clustering was then performed within each patient to summarize the tumor features into five phenotypic clusters. The corresponding local representations were input to the attention-based survival model, which generated the patient-level Risk Score.

### 3.3 Risk score for prognostic assessment of PFS

The C-index was 0.704 (95% bootstrap CI 0.637–0.768) in training, 0.663 (0.544–0.776) in the internal holdout set, and 0.706 (0.533–0.859) in SAHSYU. In univariable Cox analysis, each one-training-set-standard-deviation increase in the Risk Score was associated with PFS internally (HR 1.688, 95% CI 1.080–2.640; Wald p=0.022). In SAHSYU, the exploratory estimate was HR 6.010 (95% CI 1.170–30.877; p=0.032). In the exploratory internal adjusted model, the Risk Score estimate was HR 1.427 (95% CI 0.867–2.350; Wald p=0.162).

Univariable and exploratory adjusted Cox results for the internal validation cohort are summarized in **Table 2**. **Table 3** presents univariable SAHSYU results and indicates that the multivariable estimates were not retained because of the limited number of events.

**Table 2:** Univariable and exploratory multivariable Cox analysis in the internal validation set.

Progression-free survival				
	Univariate analysis		Multivariate analysis	
	HR (95%CI)	p*	HR (95%CI)	p*
Risk Score (per training SD)	1.688 (1.080–2.640)	0.022	1.427 (0.867–2.350)	0.162
Sex (male vs female)				
	1.700 (0.714–4.043)	0.230	1.765 (0.674–4.621)	0.247
	–	–	–	–
Age (per training SD)	1.205 (0.783–1.854)	0.397	1.008 (0.628–1.620)	0.973
T4 vs T1–3	1.886 (0.721–4.933)	0.196	2.013 (0.654–6.196)	0.223
N2 vs N1	1.410 (0.585–3.396)	0.444	1.263 (0.450–3.544)	0.657

HR, hazard ratio; CI, confidence interval.

*Two-sided coefficient p values and 95% confidence intervals are reported using the Wald framework; p<0.05 was considered statistically significant. The adjusted analysis was exploratory.

**Table 3:** Univariable Cox analysis in the exploratory SAHSYU cohort.

Progression-free survival				
	Univariate analysis		Multivariable analysis not estimated (10 events)	
	HR (95%CI)	p*	HR (95%CI)	p*
Risk Score (per training SD)	6.010 (1.170–30.877)	0.032	Not estimated	Not estimated
Sex (male vs female)			Not estimated	Not estimated
	0.775 (0.200–2.999)	0.712	Not estimated	Not estimated
	–	–	Not estimated	Not estimated
Age (per training SD)	1.145 (0.546–2.399)	0.720	Not estimated	Not estimated
T4 vs T1–3	0.409 (0.106–1.581)	0.195	Not estimated	Not estimated
N2 vs N1	0.617 (0.159–2.385)	0.483	Not estimated	Not estimated

HR, hazard ratio; CI, confidence interval.

*Two-sided Wald p values are reported. Multivariable estimates were not retained because only 10 PFS events were observed.

**Figure 2:**
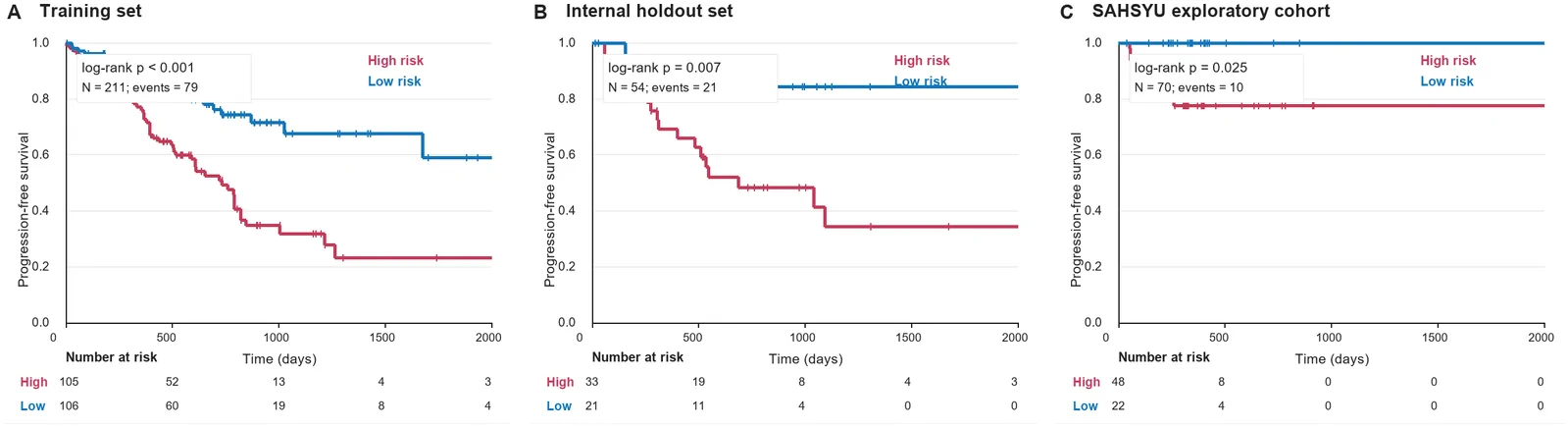
Progression-free survival according to the deep-learning risk score. Kaplan-Meier curves are shown for the training set **(A)**, internal holdout set **(B)**, and SAHSYU exploratory cohort **(C)**. Patients were assigned to high- and low-risk groups using the median Risk Score derived from the training set (0.002322592), which was then retained unchanged in the other cohorts. Tick marks indicate censored observations. Two-sided log-rank tests used the complete available follow-up. Numbers at risk are shown below each panel.

Using the training-set median cutoff, patients were classified into high- and low-risk groups and the same cutoff was applied to both validation cohorts. The Kaplan–Meier curves are shown in **Figure 2**. The observed separation showed preliminary risk-stratification potential.

### 3.4 Risk score for prognosis assessment in pathological subgroups

**Figure 3:**
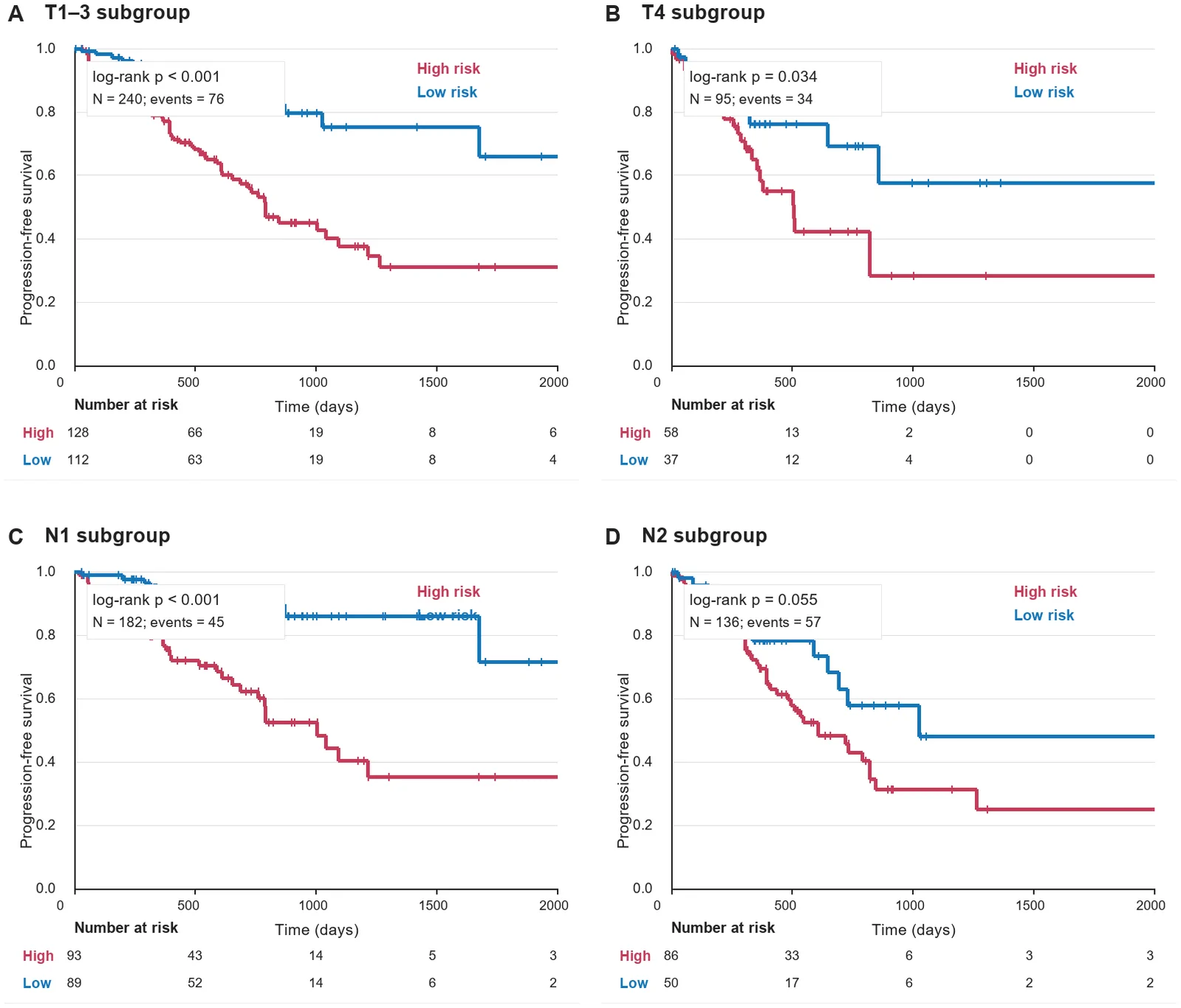
Exploratory progression-free survival analyses in pathological T- and N-category subgroups using the locked training-derived cutoff. Kaplan-Meier curves for the pooled analyzed cohorts are shown for T1-3 **(A)**, T4 **(B)**, N1 **(C)**, and N2 **(D)** subgroups. Tick marks indicate censored observations, and numbers at risk are shown below each panel. P values were calculated using two-sided log-rank tests.

Exploratory subgroup analyses are shown in **Figure 3**. In the sensitivity analysis excluding all N0 records without retraining, the training,internal, and SAHSYU cohorts contained 202/74, 48/18, and 68/10 patients/events, respectively. C-index values were 0.705, 0.647, and 0.705; fixed-training-threshold log-rank p values were <0.001, 0.014, and 0.027. The corresponding Kaplan-Meier curves are shown in **Supplementary Figure 1**.

### 3.5 Visual analysis of clustered phenotypes

**Figure 4:**
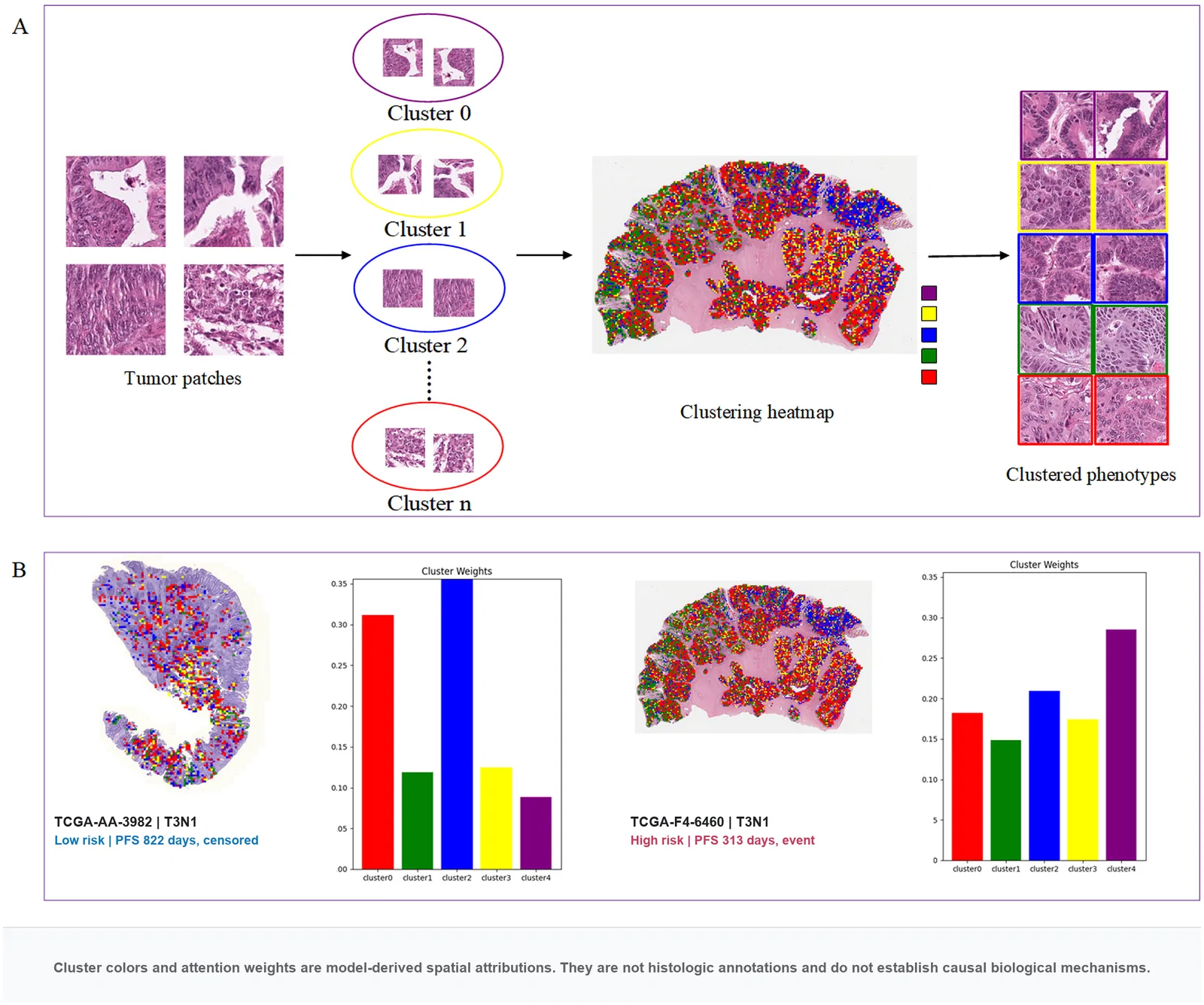
Spatial attribution of phenotypic clusters and attention weights. **(A)** Tumor tiles were assigned to computational phenotypic clusters and projected back onto the whole-slide image to generate a spatial cluster map. Representative tiles illustrate the appearance of each cluster. **(B)** Cluster maps and normalized attention weights are shown for two illustrative T3N1 TCGA cases: TCGA-AA-3982, assigned to the low-risk group (PFS, 822 days; censored), and TCGA-F4-6460, assigned to the high-risk group (PFS, 313 days; event). Cluster colors and attention weights are model-derived spatial attributions. The examples illustrate how the model weights image regions.

Attention weights quantified the relative contribution of each learned phenotypic cluster to a patient’s model output.Cluster labels and attention weights were projected onto WSIs to produce spatial heatmaps (**Figure 4**). Two illustrative patients with different PFS outcomes showed differences in phenotypic-cluster distribution and attention weighting.

### 3.6 Exploratory one-year calibration and decision-curve analysis

**Figure 5:**
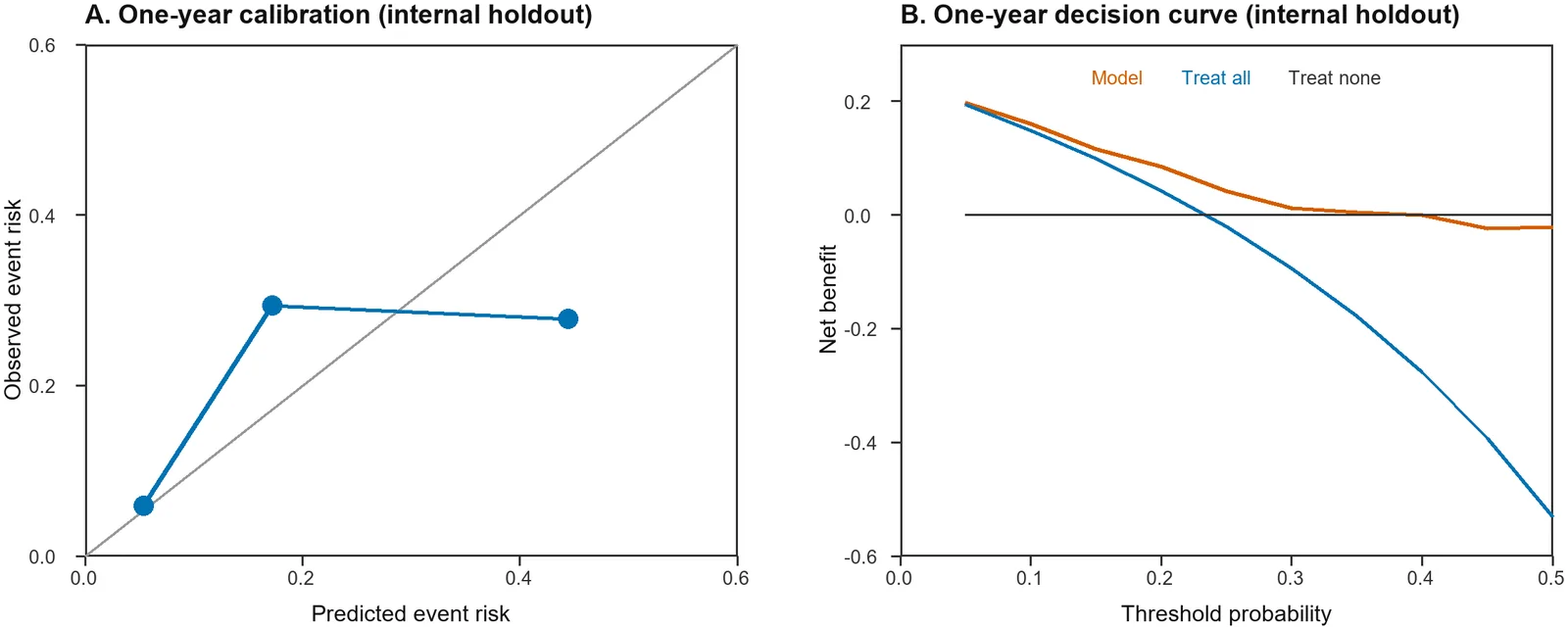
Exploratory one-year model evaluation in the internal holdout set: **(A)** calibration by thirds of predicted risk and **(B)** complete-case landmark decision-curve analysis.

In the internal holdout set, one-year calibration by thirds of predicted risk showed close agreement in the lowest-risk group (predicted 0.054 vs observed 0.059) but disagreement and non-monotonic observed risks in the middle and highest groups (**Figure 5A**). Complete-case landmark decision-curve analysis included 47 patients with observable one-year status. The model showed positive net benefit over treat-none at threshold probabilities from 0.05 to 0.35, approached zero at 0.40, and became negative above 0.40 (**Figure 5B**). Because patients censored before one year were excluded, these analyses were exploratory.

## 4 Discussion

This study integrated self-supervised contrastive learning, patient-level clustering, and attention-based aggregation to construct a locked image-derived Risk Score from routine H&E WSIs. The score showed preliminary PFS discrimination in the TCGA split and an exploratory SAHSYU evaluation, and the N0-exclusion sensitivity analysis produced similar discrimination.

The study’s contribution was the integration of annotation-free representation learning, within-patient phenotypic clustering, attention-weighted survival modeling, and spatial attribution in a stage II–III CRC workflow. Compared with supervised transfer-learning models, contrastive pretraining reduced dependence on manual labels. The present pipeline explicitly summarized within-patient phenotypic heterogeneity before attention aggregation. future same-cohort benchmarking against TNM-only Cox models, ABMIL/CLAM, TransMIL, and pathology foundation-model features is required.

Tumor heterogeneity contributes to prognostic variation in CRC (26). Five within-patient clusters were used to summarize morphological diversity, and attention weights adaptively aggregated their representations. Projection of these weights onto WSIs provided spatial model attribution.

Because the model used routine H&E slides and did not require additional staining or molecular assays, it could have relatively low incremental testing cost if implemented within an established digital-pathology workflow. A possible future workflow would apply a locked model to a quality-controlled resection WSI after routine pathological review, combine the resulting score with TNM stage and other validated factors in a multidisciplinary setting, and use the result to identify patients for prospective risk-stratified trials or surveillance research.

This study had several limitations. First, SAHSYU was small, single-center, included only 10 PFS events, and had a reverse Kaplan–Meier median follow-up of 325 days; its results are exploratory. Second, the internal adjusted model did not establish an independent association. Third, only PFS was evaluated, and OS was not validated. Finally, attention maps provided spatial attribution but not validated biological interpretation. Larger multicenter cohorts with longer follow-up, adequate events, and prospective utility assessment are required.

## 5 Conclusions

In summary, this study developed a digital pathology risk scoring model based on self-supervised learning and attention mechanisms for the exploratory assessment of progression-free survival (PFS) in stage II–III colorectal cancer (CRC). The model demonstrates preliminary discriminatory power and the ability to distinguish between risk groups; however, due to factors such as the scarcity of external events and a relatively short follow-up period, prospective, multicenter validation studies with sufficient follow-up time are required, along with calibration, benchmarking, and assessments of clinical utility.

## Data Availability

The datasets presented in this article are not readily available because The datasets used during the current study are available from the corresponding author on reasonable request. Requests to access the datasets should be directed to Xuezhi Zhou, zxzxidian@163.com.

## Supplementary material

Supplementary Figure 1 Sensitivity analysis after exclusion of patients recorded as N0. Kaplan–Meier curves are shown for the training set **(A)** internal holdout set **(B)**, and SAHSYU exploratory cohort **(C)**. Risk scores and the original training-derived cutoff (0.002322592) were retained without model retraining. Tick marks indicate censored observations. P values were calculated using two-sided log-rank tests. Numbers at risk are shown below each panel.
